# Effect Of Cariprazine On Metabolic Parameters In Patients Living With Major Depressive Disorder: A Systematic Review And Meta-Analysis

**DOI:** 10.1192/j.eurpsy.2026.10422

**Published:** 2026-07-31

**Authors:** M. Pompili, M. Cifrodelli, A. V. Mammoliti, F. Formica, R. Iannazzo, R. S. McIntyre, I. Berardelli

**Affiliations:** 1 Department of Neurosciences, Mental Health and Sensory Organs, Faculty of Medicine and Psychology, Suicide Prevention Centre, Sant’Andrea Hospital, Sapienza University of Rome, Via di Grottarossa, 1035, 00189; 2 Department of Human Neurosciences, Sapienza University of Rome; 3 Psychiatry Residency Training Program, Faculty of Medicine and Psychology, Sapienza University of Rome, Psychiatry Unit, Sant’Andrea Hospital,00185, Rome, Italy; 4 Department of Psychiatry, University of Toronto, Department of Pharmacology and Toxicology, University of Toronto, Toronto, Ontario, Canada

## Abstract

**Introduction:**

Cariprazine is a second-generation antipsychotic approved for treating schizophrenia, acute manic or mixed episodes associated with bipolar I disorder and as adjunctive treatment in major depressive disorder. Antipsychotic treatment is often associated with metabolic alterations including weight gain, dyslipidemia, and increased risk of type 2 diabetes and cardiovascular disease.

**Objectives:**

We performed a systematic review and meta-analysis with the overarching aim of synthesizing study results that describe the effect of cariprazine on glucose and lipid homeostasis as well as weight in persons living with major depressive disorder.

**Methods:**

Six meta-analyses focused on patients with major depressive disorder (MDD), evaluating changes in weight, cholesterol, and glucose for both Cariprazine 1.5 mg and 3 mg.

**Results:**

Cariprazine showed a limited impact on the metabolic profile in MDD. Particularly, weight gain was statistically significant in both doses, but clinically limited (<1 kg), with a percentage of subjects reporting an increase ≥7% of body weight less than 5%, confirming the relative metabolic safety of the drug also in this population. A minimum increase in fasting glucose at 3 mg was observed.

**Conclusions:**

This meta-analysis suggests that cariprazine, although associated with modest weight gain in MDD, and with a minimum increase in fasting glucose at 3 mg without significant alterations in lipid and glycemic profiles, shows a favorable metabolic profile compared to other atypical antipsychotics.

**Disclosure of Interest:**

None Declared

